# The First Report of the Acrotretoid Brachiopod *Hadrotreta* from the Tsinghsutung Formation Cambrian (Series 2, Stage 4), Guizhou, South China

**DOI:** 10.3390/biology12081083

**Published:** 2023-08-03

**Authors:** Buqing Wei, Yuan Wang, Xinglian Yang, Weiyi Wu

**Affiliations:** 1 College of Resources and Environmental Engineering, Guizhou University, Guiyang 550025, China; bqwei2017@163.com (B.W.); circle81906@163.com (Y.W.); 2 Guizhou Research Center for Palaeontology, Guiyang 550025, China; 3 Key Laboratory of Karst Georesources and Environment, Ministry of Education, Guiyang 550025, China; 4 College of Resources and Environmental Engineering, Guizhou Institute of Technology, Guiyang 550003, China; wuweiyi740306@163.com

**Keywords:** *Hadrotreta*, palaeogeography, Tsinghsutung Formation, Cambrian, south China

## Abstract

**Simple Summary:**

A number of well-preserved fossils of *Hadrotreta* were found in the Tsinghsutung Formation of Cambrian Series 2, Stage 4 in Jianhe, Guizhou, south China by etching rocks with 2–3% acetic acid. This is the first report of *Hadrotreta* in south China. According to the global palaeogeographical distribution of *Hadrotreta* shows an expanding trend from the Cambrian Age 4 to the Miaolingian Epoch, and this genus was mainly found at low latitudes. In the Cambrian Epoch 2, Age 4, *Hadrotreta* only appeared in south China and the Laurentia palaeocontinent, and was mostly associated with deep-water continental shelf environments. Later, *Hadrotreta* expanded its distribution to become virtually cosmopolitan during the Miaolingian Epoch and is mostly preserved in shallow-water platform environments.

**Abstract:**

*Hadrotreta* is a worldwide acrotretoid brachiopod reported from the Cambrian Series 2 to Miaolingian. Here, a number of well-preserved fossils of *Hadrotreta*, identified as *Hadrotreta* cf. *H*. *timchristiorum*, were found in the *Protoryctocephalus arcticus* Zone of the Tsinghsutung Formation of Cambrian Series 2, Stage 4 in Jianhe, Guizhou, south China. This is the first report of *Hadrotreta* in China, which enriches its global palaeogeographical distribution. *Hadrotreta* is very similar to acrotretoids such as *Kostjubella*, *Vandalotreta*, *Linnarssonia*, and *Eohadrotreta*. It differs from them with its well-developed ventral boss-like apical process, apical pits, and dorsal median sulcus. In view of the palaeogeography of *Hadrotreta*, this genus was mainly distributed in low-latitude regions. *Hadrotreta* was only found in south China and Laurentia during the Cambrian Age 4, then expanded its distribution to other regions such as Siberia, Baltica, the Kazakh Terranes, the Far East, and Gondwana Pange during the Miaolingian Epoch. *Hadrotreta* seems to have shifted from deeper water to shallow-water environments during the period from the Cambrian Series 2 to the Miaolingian.

## 1. Introduction

Acrotretoid brachiopods first appeared in the Cambrian Epoch 2 Age 3 (late Atdabanian), and diversified rapidly during the Miaolingian. They later significantly increased their taxonomic diversity in the early Ordovician, before diminishing and declining and finally becoming extinct in the Devonian [1,2,3,4,5,6,7]. However, their origin, earliest evolution, and ontogeny are still poorly understood. Current knowledge indicates that, during the Cambrian, acrotretoids expanded rapidly from 8 genera in the Cambrian Epoch 2 to 37 genera in the Miaolingian and 23 genera in the Furongian [2,4,6]. Acrotretoids found in the Cambrian Epoch 2 include *Eohadrotreta*, *Linnarssonia*, *Prototreta*, *Vandalotreta*, *Kuangshanotreta*, *Hadrotreta*, *Kostjubella*, and *Palaeotreta* [2,3,4,6,8,9]. The continuous progress of scanning imaging technology in recent years has enabled the study of the early evolution of brachiopods through examinations of their microstructure [5,10,11,12,13]. Some acrotretids are well preserved in three dimensions and often reveal fine details of their ornamentation and delicate shell structures [11,13,14,15]. Zhang et al. [5,12,13] not only studied the relationship between the ontogenetic stage of *Eohadrotreta*, their distribution of epidermal cells, and the attachment area of their soft body, but also explored the systematic relationship between *Eohadrotreta* and other lingulid brachiopods, providing an excellent basis for further research on the early origin and expansion of brachiopods. As one of the oldest and most cosmopolitan acrotretoids, *Hadrotreta* has been reported from the Cambrian Series 2 to Miaolingian in Nevada, California, Pennsylvania, Canada, Turkestan, Uzbekistan, Novaya Zemlya, Kazakhstan, the Far East, Australia, the Himalayas, and Mexico [16,17,18,19,20,21,22,23,24,25,26,27,28]. In addition, *Hadrotreta*? sp. also occurs in the Cerro Pelado Formation of the Middle–Upper Cambrian (Miaolingian–Furongian) boundary beds at the Cerro Pelado section in western Argentina [29]. However, *Hadrotreta* had not been found in south China until now, although abundant acrotretoids such as *Eohadrotreta*, *Kuangshanotreta*, *Linnarssonia*, and *Palaeotreta* have been reported [4,6,7,8].

Recently, a number of well-preserved specimens of *Hadrotreta* fossils, identified as *Hadrotreta* cf. *H*. *timchristiorum*, were collected from the middle and upper Tsinghsutung Formation of Cambrian Series 2, Stage 4 in Jianhe, Guizhou, south China. This paper is the first report of *Hadrotreta* from China. Its detailed morphology and internal structures show that *Hadrotreta* is very similar to other genera such as *Eohadrotreta*, *Vandalotreta*, *Kostjubella*, and *Linnarssonia*. This paper expands the global palaeogeographical distribution and summarizes the spatiotemporal distribution of Cambrian *Hadrotreta*.

## 2. Materials and Methods

### 2.1. Geological Setting

The study area was located in Jianhe County, 240 km to the southeast of Guiyang City, and belonged to the transition slope area between the Yangtze platform and Jiangnan basin [30]. The Cambrian strata in the Jianhe area are widely distributed, including the Niutitang, Jiumengchong, Bianmachong, Balang, Tsinghsutung, Kaili, Jialao, and Loushanguan formations in ascending order [31,32].

**Figure 1:**
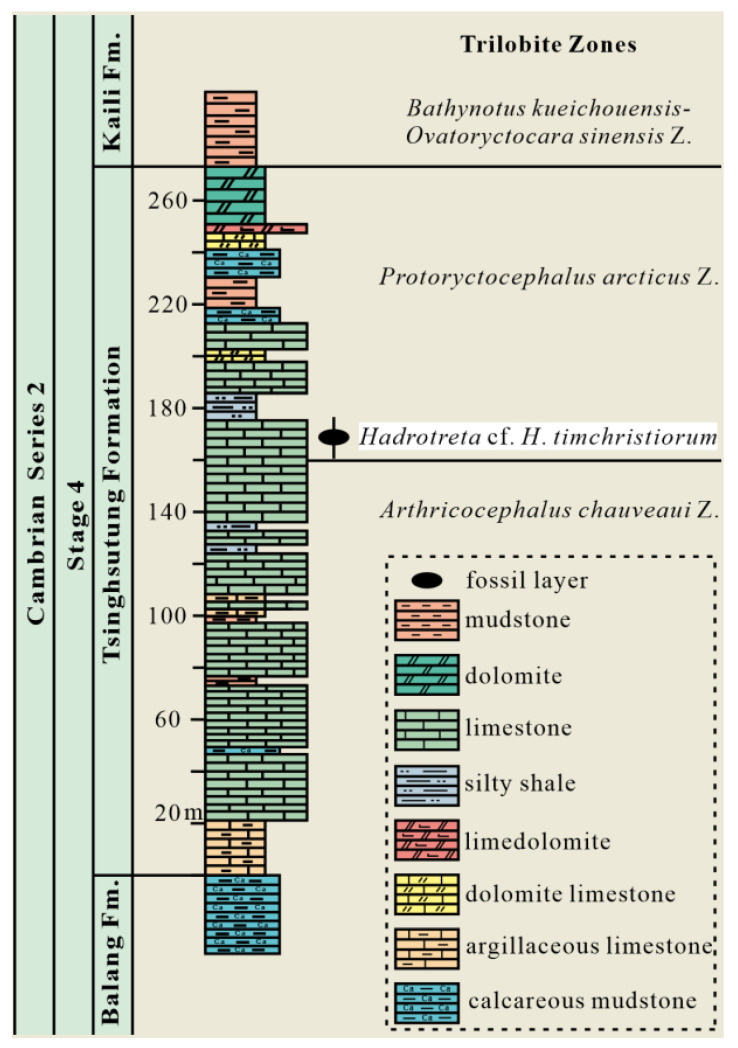
Stratigraphic columns of the Tsinghsutung Formation at the Songshan section in Jianhe County, Guizhou Province, south China, showing the stratigraphic horizon of acrotretoid *Hadrotreta* cf. *H*. *timchristiorum*.

The studied section is located in Songshan, Balang Village, Jianhe County, and is adjacent to the Wuliu-Zengjiayan section, the Global Stratotype Section of the Cambrian Miaolingian [31,32]. The Tsinghsutung Formation of the Songshan section is in conforming contact with the overlying Kaili Formation and underlying Balang Formation, and the sedimentary age is assigned to the Cambrian Epoch 2, Age 4, with a lithological composition of limestone, dolomite, and mudstone. The Tsinghsutung Formation has a thickness of about 272.2 m. It is divided into a lower portion composed of greyish-grey, medium-thick limestone, an upper part comprising a medium-thick layer, a thin layer of limestone and a thick silty muddy layer, and a top layer of grey-black dolomite (Figure 1) [31,32,33]. Zhang et al. considered the Tsinghsutung Formation of the Songshan section in Jianhe to have been deposited in a low-energy, deep-water shelf–shelf margin environment [34]. In terms of biostratigraphy based on trilobites, the Tsinghsutung Formation in the Songshan section of Jianhe is divided into an *Arthricocephalus chauveaui* zone in the lower part and a *Protoryctocephalus arcticus* zone in the middle-to-top part. Here, we report the collection of *Hadrotreta* cf. *H*. *timchristiorum* in the *Protoryctocephalus arcticus* zone.

### 2.2. Hadrotreta Material and Specimen Visualization

The specimens from the limestones of the Tsinghsutung Formation in the Songshan section were etched in a solution of 2–3% acetic acid, as proposed by Jeppsson et al. [35]. The samples obtained by sifting, washing, and drying were then selected under a stereomicroscope to obtain the fossil material. Scanning electron microscope (SEM) images of coated fossils were taken with EVO18 and SU3500 with 15–20 kV and 70–80 Pa at the Nanjing Institute of Geology and Palaeontology, Chinese Academy of Sciences. For some specimens, SEM imaging was carried out at 20 kV with a COXEM-30 from Guizhou University.

## 3. Results and Discussion

### 3.1. Systematic Palaeontology

Subphylum Linguliformea Williams et al., 1996 [36].

Class Lingulata Gorjansky and Popov, 1985 [37].

Order Acrotretida Kuhn, 1949 [38].

Superfamily Acrotretoidea Schuchert, 1893 [39].

Family Acrotretidae Schuchert, 1893 [39].

Genus *Hadrotreta* Rowell, 1966 [40].

Type Species: *Acrotreta primaea* Walcott, 1902; lower to middle Cambrian (Stage 4 to Wuliuan) Pioche Shale, Nevada, USA.

Diagnosis. Shell transversely oval or subcircular, ventribiconvex, shell ornamented by fine growth lines. Ventral valve low subconical; pseudointerarea flat to gently concave, catacline to gently procline, divided medially by shallow intertrough; external pedicle foramen small, subelliptical, immediately posterior of apex; apical process boss-like and entirely anterior of apex of valve, limited laterally by *vascula lateralia*; and apical pits well-developed, immediately lateral of pedicle tube. Dorsal valve gently convex, anteriorly with broad, shallow median sulcus; dorsal pseudointerarea orthocline to gently anacline, divided by median groove into two propareas; and cardinal muscle scars relatively long. For detail, see Rowell [40] (p. 12).

Discussion. *Hadrotreta* was first established by Rowell [40]. The type species is *Acrotreta primaea*, which was reported in the lower to middle Cambrian Pioche Shale of Nevada, USA, by Walcott [41], who placed in the genus *Acrotreta* base on the character of pseudointerarea, which was similar to *Acrotreta attenuata* [41,42]. *Hadrotreta* was then established by Rowell [40], who revised the classification based on the presence of a short pseudointerarea, a lower median ridge rather than a leaf-like septum, a short-small pedicle foramen, and the shape and location of the apical process.

The morphological characteristics of *Hadrotreta* are roughly similar to *Kostjubella*, *Vandalotreta*, *Linnarssonia*, and *Eohadrotreta*, although with important differences (Table 1). For example, *Hadrotreta* and *Kostjubella* have a well-developed shallow median sulcus and median ridge of the dorsal valve, the difference being that the latter ventral valve pseudointerarea are catacline to slightly apsacline with a deeper intertrough, and the maximum height anterior to the umbo and apical process is confined to a boss-like raised area surrounding the internal foramen (Table 1) [9,43,44]. When comparing *Hadrotreta* with *Vandalotreta* [45], the ventral valve boss-shaped apical process of the latter does not fill the apex, and the dorsal median sulcus and median ridge are vestigial or weakly developed (Table 1) [46]. The genus *Linnarssonia* [47] differs from *Hadrotreta* in that the ventral valve apical pits are not developed, the pedicle foramen is not enclosed in a larval shell, the ventral pseudointerarea are catacline to procline, the dorsal pseudointerarea is weakly developed, the cardinal muscle scars are arranged more closely, the dorsal median ridge is high and short, and the dorsal median buttress is well developed (Table 1) [7]. The difference between *Eohadrotreta* [8] and *Hadrotreta* is that the ventral valve of the former lacks a well-developed apical process and apical pits, and the dorsal median sulcate is absent (Table 1).

At present, the genus *Hadrotreta* contains the following species: *H*. *primaea* [18,20,28,40,41,42,50], *H*. *taconica* [25], *H*. *timchristiorum* [23], *H*. *djagoran* [51], *H*. *fragilis* [19], *H*. *extentusa* [17,21], *H*. *pallialis* [16], *?H*. *primaea* [52], *Hadrotreta* sp. [22,24,25], *Hadrotreta rara*? [27], and *Hadrotreta*? sp. [29]. *H*. *taconica* differs from the type species by the ventral apical pits anterior to the pedicle foramen and the lack of a boss-like termination to the dorsal median ridge. It differs from *H*. *trimchristiorum* by a larger dorsal pseudointerarea and a deeper ventral intertrough. *H*. *timchristiorum* is more similar to *H*. *primaea*; both have a well-developed, boss-like apical process. *H*. *timchristiorum* differs from the type species mainly in having a narrow and shallow ventral intertrough, as well as a shorter and less-developed dorsal pseudointerarea, and a smaller and shorter dorsal cardinal muscle field. *H*. *djagoran* was revised to *Vandalotreta djagoran* by Holmer et al. [46].

Occurrence. Cambrian Series 2 to Miaolingian; the United States (Nevada, California, and Pennsylvania), Canada, Mexico, Siberia, Britain, Kazakhstan, Far East, south China, India, and Australia.

**Figure 2:**
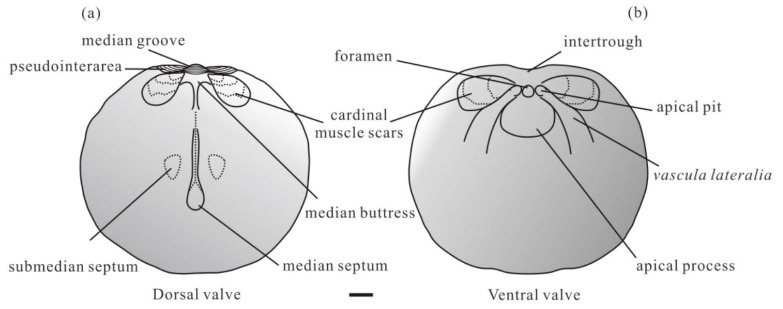
Schematic reconstructions of the dorsal valve (**a**) and ventral valve (**b**) interior, Scale bar = 100 µm.

**Figure 3:**
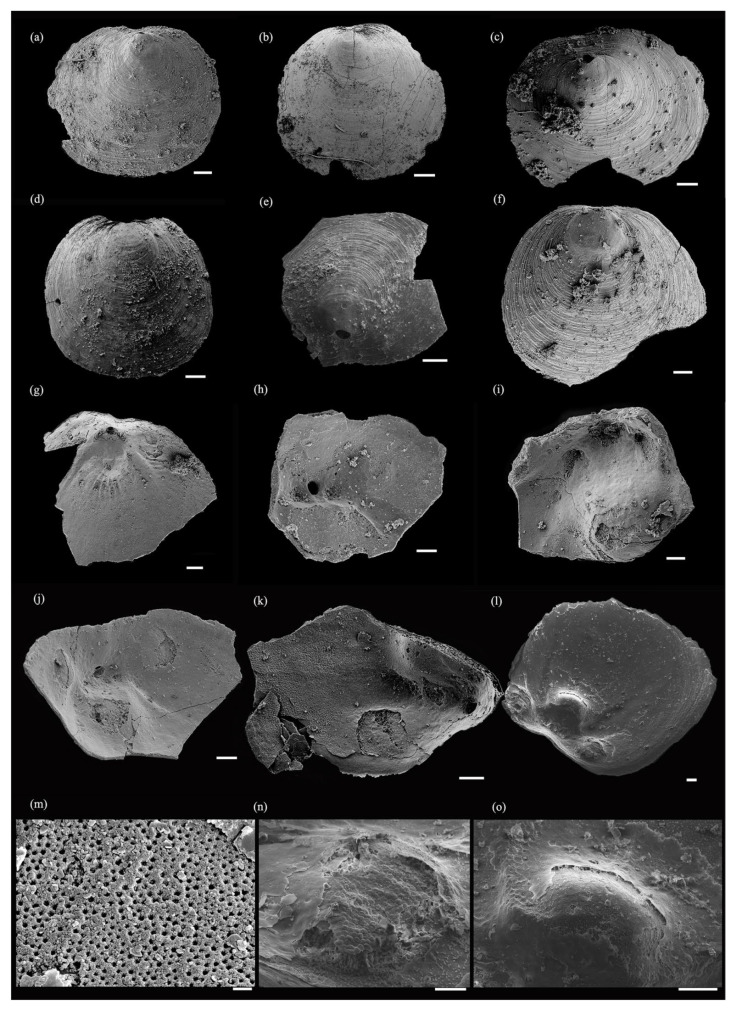
Ventral valve of *Hadrotreta* cf. *H*. *timchristiorum* from the Tsinghsutung Formation of Cambrian at Songshan section in Jianhe County, Guizhou Province, China. (**a**–**f**) Ventral valves exterior view; (**a**) oblique lateral view, sample JHQH-163-9; (**b**) oblique lateral view, sample JHQH-169-20; (**c**) oblique lateral view, sample JHQH-169-40; (**d**) oblique lateral view, sample JHQH-169-35; (**e**) oblique posterior view, sample JHQH-169-114; (**f**) oblique lateral view, sample JHQH-169-28; (**g**–**o**) ventral valves interior view; (**g**) ventral valve interior, sample JHQH-169-21; (**h**) ventral valve interior side view, sample JHQH-163-16; (**i**) ventral valve interior side view, sample JHQH-163-901; (**j**) ventral valve interior side view, sample JHQH-163-50; (**k**) ventral valve interior side view, sample JHQH-169-32; (**l**) ventral valve interior side view, sample JH-172-04-1; (**m**) pitted micro-ornament of larval shell, a dorsal umbonal region showing larval shell (**b**); (**n**) enlarged view of ventral cardinal muscle (**l**); and (**o**) enlarged view of ventral apical process (**l**). Scale bars = 100 µm (**a**–**l**), 2 µm (**m**), and 50 µm (**n**,**o**).

**Figure 4:**
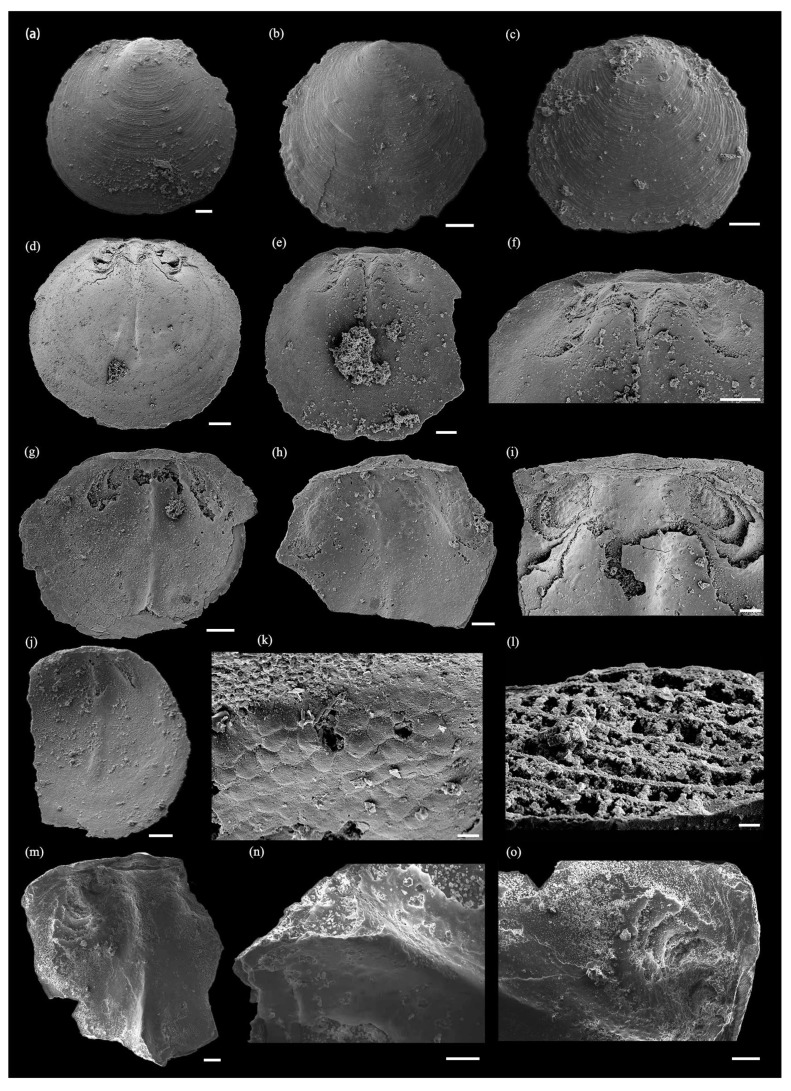
Dorsal valve of *Hadrotreta* cf. *H*. *timchristiorum* from the Tsinghsutung Formation of Cambrian at Songshan section in Jianhe County, Guizhou Province, China. (**a**–**c**) Dorsal valves exterior view; (**a**) oblique lateral view, sample JHQH-163-10; (**b**) oblique lateral view, sample JHQH-163-11; (**c**) oblique lateral view, sample JHQH-163-34; (**d**–**o**) dorsal valves interior view; (**d**) dorsal interior view, sample JHQH-163-12; (**e**) dorsal interior view, sample JHQH-169-141; (**f**) enlarged view of interarea and cardinal muscle fields (**e**); (**g**) dorsal interior view, sample JHQH-169-161; (**h**) oblique lateral view of dorsal interior, sample JHQH-169-60; (**i**) enlarged view of cardinal muscle fields, sample JHQH-163-58; (**j**) oblique lateral view of dorsal interior, sample JHQH-163-38; (**k**,**l**) enlarged view of cardinal muscle and media groove (**d**), JHQH-163-12; (**m**) dorsal interior view, sample JH-172-05; and (**n**,**o**) enlarged view of the median septum, median buttress, and cardinal muscle (**m**), JH-172-05. Scale bars = 100 µm (**a**–**j**,**m**,**o**), 10 µm (**k**,**l**), and 50 µm (**n**).

*Hadrotreta* cf. *H*. *timchristiorum* Popov et al. 2015 (Figure 2, Figure 3 and Figure 4).

Material. Ten dorsal valves and twelve ventral valves from acid-resistant residues of limestone samples from the Tsinghsutung Formation, Songshan section in the Guizhou Province, China.

Description. Shell transversely oval or subcircular, ventribiconvex (Figure 2); shell ornamented by concentric growth lines. The ventral valve is conical, the ratio of the ventral valve length to width is approximately 0.8945 (Table 2), and the concentric sculpture on the shell surface is strongly developed, gradually becoming sparse from the posterior to the anterior (Figure 3a–f). The apex of the ventral shell is slightly convex to subconical, and the vertex is in the middle to the anterior of the shell (Figure 3a,b,d,f). The posterior edge of the ventral shell is short and rounded, the beak slightly protrudes forward, and the larval shell is covered by hemispherical pits (Figure 3c,f,m). The pedicle tube with a narrow and shallow intertrough touches the larval shell posterior (Figure 3e,g,j). A high, broadly boss-like apical process is well preserved in the ventral interior, with a lamellar structure and column structure in the transverse section (Figure 3i,k,l,o). The rounded pedicle foramen is situated in the posterior part of the apical process and apical pits are situated lateral to and slightly anterior of the pedicle tube (Figure 3h–j); cardinal muscles are situated lateral to the *vascula lateralia*, at the lateral margins of the ventral pseudointerarea, with a well-preserved honeycomb-like cell structure (Figure 3j,l,n). The ventral visceral region and the *lateral cateria* are well-preserved, and the *lateral cateria* are relatively short. Five fines *lateral cateria* extend from the apical process to the anterior, which is situated between the normal *lateral cateria* (Figure 3g).

The dorsal valve is subcircular, and the ratio of the length to width is approximately 0.9433 (Table 2), with a slightly convex apex. The dorsal shell is ornamented with fine, concentric ridges, separated by a broad, shallow anterior sulcus (Figure 4a–c), and the shell appears with a lamellar structure. The dorsal pseudointerarea is narrowly triangular, orthocline to anacline with a column-structured median groove (Figure 4l), the ratio of the pseudointerarea width to shell width is nearly 0.3761, and the ratio of the median groove width to the pseudointerarea is about 0.4226 (Table 2). A triangular median buttress is beneath the median groove, connected with the spindle-like median septum (Figure 4d,g,j,m), and the ratio of the median septum length to the dorsal valve is about 0.6862 (Table 2). The submedian septum is situated in the lateral median septum. The dorsal cardinal muscle is a long ellipse, situated between the lateral of the median septum and extending to the edge of the dorsal valve from the pseudointerarea (Figure 4e,f,h,i), with a well-preserved honeycomb-like cell structure (Figure 4k), the same as in the cardinal muscles in the ventral valve (Figure 3n). The ratio of the pseudointerarea length to the dorsal valve length is about 0.2211 (Table 2).

Comparison. The majority of acrotretoid brachiopods from the Tsinghsutung Formation belong to *Hadrotreta*, and have been identified as such based on the oval pedicle foramen on the outside of the larval shell, the boss-like apical process of the ventral valve, the presence of deep apical pits lateral to the internal pedicle tube, and the low, forked dorsal median ridge. *Hadrotreta* cf. *H*. *timchristiorum* differs from the type species, *H*. *primaea*, from the Pioche Shale of the Great Basin, by a weakly developed median ridge system in the dorsal valve and apical pits laterally located in the internal foramen. *Hadrotreta* cf. *H*. *timchristiorum* is distinguished from *H*. *taconica* in Canada by a larger dorsal pseudointerarea, larger dorsal muscle scars, a less protruding apical process, and a more well-defined ventral intertrough. The material in the Tsinghsutung Formation is similar to that of *H*. *timchristiorum* in the Parahio Formation, but differs in having a relatively well-developed ventral apical process and *vascula lateralia* (Figure 3g,l), with well-developed dorsal cardinal muscle fields (Figure 4f,i,k,o).

### 3.2. Palaeogeographical Implication of Hadrotreta

**Figure 5:**
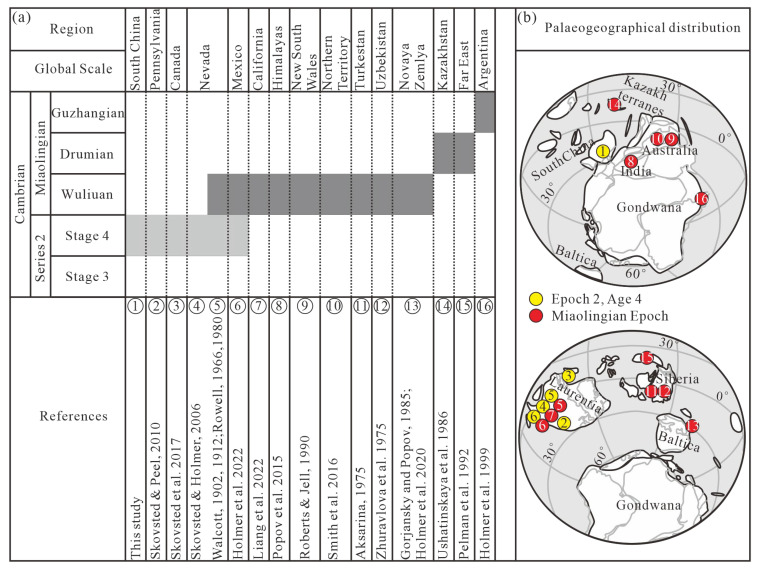
Maps of stratigraphic occurrence, geographical, and palaeogeographical distribution of the acrotretoid brachiopod *Hadrotreta* in the Cambrian. (**a**) Stratigraphic occurrence and geographical distribution of the acrotretoid brachiopod *Hadrotreta* in Cambrian (Cambrian Stage 4 and Miaolingian are marked by light grey and dark grey shades, respectively); and (**b**) palaeogeographical distribution of the acrotretoid brachiopod *Hadrotreta*, the palaeogeographical map modified from Holmer et al. [27]. The numbers such as ①–⑩ of (**a**,**b**) show the distribution of *Hadrotreta* [16,17,18,19,20,21,22,23,24,25,26,27,28,29,37,40,41,42,52].

At present, *Hadrotreta* is currently known to be from Nevada [18,40,41,42,52], California [28], Pennsylvania [22], Canada [25], the Himalayas [23], Australia [20,24], Turkestan [16], Uzbekistan [17], Novaya Zemlya [26,37], Kazakhstan [19], Argentina [29], the Far East [21], and Mexico [27], from sites with a geological age ranging from Cambrian Age 4 to the Miaolingian Epoch (Figure 5a). *Hadrotreta* occurred in Pennsylvania, Nevada, Canada, and Mexico during the Cambrian Age 4 (Figure 5a), where it was located in Laurentia (Figure 5b). However, during the Cambrian Miaolingian Epoch, *Hadrotreta* also appeared in other regions such as Siberia [16,17], Baltica [26,37], Kazakh Terranes [19], the Far East [21], and Gondwana Pangae [20,23,24,29], indicating that the palaeogeographical distribution of *Hadrotreta* rapidly expanded in this period (Figure 5b). This paper is the first report of *Hadrotreta* cf. *H*. *timchristiorum* from the Cambrian in south China, and the specimens were mainly collected from the *Protorytocephalus arcticus* zone of the Tsinghsutung Formation in Cambrian Age 4, Guizhou, providing new information and expanding the temporal and spatial distribution of *Hadrotreta* (Figure 5). In addition, according to the global palaeogeographical distribution of *Hadrotreta* from Cambrian Age 4 to Miaolingian Epoch, it is shown that the genus is mainly found at low latitudes (Figure 5b).

The evidence from south China (this study) and Canada indicates that *Hadrotreta* seems to have lived in deep-water outer shelf environments during the Cambrian Age 4 (Table 3) [13,25]. Materials from Canada also show that *Hadrotreta* was also found in shallow-water environments of the inner shelf (Table 3) [25]. However, during the Miaolingian, *Hadrotreta* lived in shallow-water environments (Table 3) [18,20,23,28], which shows a wide ecological range for *Hadrotreta*. The global palaeogeographical distribution of *Hadrotreta* shows an expanding trend from the Cambrian Age 4 to the Miaolingian Epoch. Moreover, during the Cambrian Age 4, *Hadrotreta* seems to have lived in deeper-water environments. In contrast, it was in relatively shallow-water environments in the Miaolingian Epoch (Table 3).

## 4. Conclusions

Well-preserved *Hadrotreta* fossils from the Tsinghsutung Formation of the Cambrian Series 2, Stage 4 at the Songshan section, Jianhe, Guizhou, China, identified as *Hadrotreta* cf. *H*. *timchristiorum*, are the first of this genus to be reported in China, increasing its known distribution. *Hadrotreta* is similar to *Kostjubella*, *Vandalotreta*, *Linnarssonia*, and *Eohadrotreta*, but the former has a well-developed ventral boss-like apical process, apical pit, shallow intertrough, and dorsal median sulcus, which separate it from the other genera.

According to its palaeogeographical distribution, *Hadrotreta* is mainly found at low latitudes. In the Cambrian Epoch 2, Age 4, *Hadrotreta* only appeared in south China and the Laurentia palaeocontinent, and was mostly associated with deep-water continental shelf environments. Later, *Hadrotreta* expanded its distribution to become virtually cosmopolitan during the Miaolingian Epoch and is mostly preserved in shallow-water platform environments.

## Figures and Tables

**Table 1:** Morphological comparison of characteristics of *Hadrotreta* with similar genera (*Kostjubella*, *Vandalotreta*, *Linnarssonia*, and *Eohadrotreta*).

Genus	*Hadrotreta*	*Kostjubella*	*Vandalotreta*	*Linnarssonia*	*Eohadrotreta*
**Ventral valve**	Shell transversely oval, ventral valve moderately convex to subconical with maximum height at the umbo or beak	Ventral valve strongly convex in lateral profile with maximum height anterior to the umbo	Shell transversely oval to subcircular with maximum height at the umbo or beak	Shell subcircular to transversely oval, and ventral valve convex to low subconical	Shell subcircular to transversely oval, and ventral valve convex to low subconical
**Ventral pseudointerarea**	Catacline to gently procline and divided medially by shallow intertrough	Catacline to slightly apsacline, narrow and divided by deeper intertrough	Procline to catacline, poorly defined laterally and divided by intertrough	Catacline to procline, rarely apsacline and divided by shallow intertrough	Gently procline and with shallow to vestigial intertrough
**Apical pit**	Present	Present	Present	Present	Vestigial to absent
**Apical process**	Boss-like and anterior to the internal foramen	Boss-like and raised area surrounding the internal foramen	Boss-like and thickening anterior to internal foramen but not filled apex	High, boss-like, and anterior to the foramen	Vestigial to absent
**Dorsal valve**	Gently convex and with broad, shallow sulcus	Gently convex and with shallow sulcus	Weakly convex and absent sulcus	Gently convex and absent sulcus	Gently convex and absent sulcus
**Dorsal pseudointerarea**	Narrowly triangular and with orthocline to anacline	Low	Short	Vestigial and undivided	Narrowly triangular and with orthocline
**Median groove**	Shallow median groove	Lenticular median groove	Broadly triangular median groove	Broadly median groove	Shallow median groove
**Median ridge**	Low	Strong, low to subtriangular	Vestigial	High	Well-developed submedian ridge
**Median buttress**	Well-developed and posteriorly by a low median ridge	Well-developed and posteriorly discontinuous medially	Well developed	Low	Well developed
**Reference**	Rowell, [40]	Percival & Kruse, [9]; Popov et al. [43] and Holmer et al. [48]	Streng, [49]	Holmer & Popov, [3] and Duan et al., [7]	Li and Holmer, [8]

**Table 2:** Main dimensions and ratios of the dorsal and ventral valve structure (size unit: mm).

**D**	**L**	**W**	**Lp**	**Wp**	**Ls**	**Wg**	**Lc**	**Wc**
**N**	8	8	18	15	10	16	12	9
**S**	0.3939	0.4323	0.0121	0.0977	0.2706	0.0425	0.0775	0.1368
**X**	0.9636	1.0285	0.0434	0.4275	0.7708	0.1792	0.2759	0.6180
**max**	1.8257	2.0183	0.0670	0.6610	1.2600	0.2900	0.4180	0.8570
**min**	0.5184	0.5494	0.0229	0.2767	0.4565	0.1401	0.1729	0.4314
**D**	**L/W**	**Wp/W**	**Lp/Wp**	**Wg/Wp**	**Lc/L**	**Wc/W**	**Ls/L**	
**N**	8	3	15	15	4	3	6	
**S**	4.16	4.94	1.87	7.43	3.44	2.95	4.66	
**X**	0.9433	0.3761	0.0995	0.4226	0.2211	0.4455	0.6862	
**max**	0.9787	0.4263	0.1249	0.5305	0.2696	0.4663	0.7560	
**min**	0.9046	0.3275	0.0638	0.2953	0.1961	0.4246	0.6200	
**V**	**L**	**W**	**Lf**	**Wf**	**L/W**	**Lf/L**	**Wf/W**	**Lf/Wf**
**N**	6	6	8	8	6	2	2	8
**S**	0.2974	0.3301	0.0149	0.0161	0.0552	0.0029	0.0113	0.1412
**X**	0.4663	0.5223	0.0526	0.0649	0.8945	0.0584	0.0726	0.8137
**max**	1.5843	1.7599	0.0879	0.1078	0.9929	0.0613	0.0839	1.0956
**min**	0.7284	0.8019	0.0372	0.0506	0.8058	0.0554	0.0612	0.5886

Abbreviations: D—dorsal valve; V—ventral valve; N—number of specimens; X—mean; S—standard deviation; min—minimum observed size; and max—maximum observed size. In other abbreviations, all measurements are in millimetres. L—length of the valve; Lp—length of pseudointerarea, Ls—length of the median septum; Lc—length of cardinal muscle scar; Lf—length of foramen; W—width of the valve; Wp—width of pseudointerarea; Wc—width of cardinal muscle scar; Wg—width of median groove; and Wf—width of the foramen.

**Table 3:** The classification, diversity, age, and sedimentary environment of *Hadrotreta* Rowell, 1966 (this table only compares the published confirmed species, however, undetermined species, similar species, and doubted species are not discussed).

Species	Formation	Region	Ages	Biostratigraphy	Sedimentary Environment	Reference
*Hadrotreta Primaeva Primaeva Hadrotreta Primaeva minor*	Pioche shale	Nevada, USA	Age 4 to Wuliuan	Late *Bonnia–Olenellus* Zone and Pre-*Albertella* Zone	Sea margin of the Carbonate platform	Rowell, [18,40]
*Hadrotreta primaea*	Cadiz Fm.	California, USA	Wuliuan	*Olenellus multinodus* Subbiozone of the *Bonnia–Olenellus* Zone	Open shallow subtidal	Liang et al. [28]
*Hadrotreta taconica*	Forteau Fm.	Canada	Age 4	*Bonnia–Olenellus* Zone	The inner shelf is in shallow water and the distal shelf setting in deep water	Skovsted et al. [25]
*Hadrotreta primaeva*	Coonigan Fm.	New South Wales, Australia	Wuliuan	Occurrence of *Pagetia*	Edge of a shallow shelf	Roberts and Jell, [20]
*Hadrotreta timchristiorum*	Parahio Fm.	Himalayas, India	Wuliuan	*Orycticephalus salteri*–*Paramecephalus–Defossu* Zone	Seaward-facing shelf setting	Popov et al. [23]

## Data Availability

Not applicable.
